# Lost to the NHS: a mixed methods study of why GPs leave practice early in England

**DOI:** 10.3399/bjgp16X683425

**Published:** 2016-01-07

**Authors:** Natasha Doran, Fiona Fox, Karen Rodham, Gordon Taylor, Michael Harris

**Affiliations:** Department for Health, University of Bath, Bath; senior research fellow, South West Academic Health Science Network (SWAHSN), Exeter.; NIHR Collaborations for Leadership in Applied Health Research and Care (CLAHRC) West, University of Bristol, Bristol.; Staffordshire University, Stoke-on-Trent.; Department for Health, University of Bath, Bath.; Department for Health, University of Bath, Bath.

**Keywords:** burnout, general practice, job satisfaction, professional autonomy, qualitative research, workload

## Abstract

**Background:**

The loss of GPs in the early stages of their careers is contributing to the GP workforce crisis. Recruitment in the UK remains below the numbers needed to support the demand for GP care.

**Aim:**

To explore the reasons why GPs leave general practice early.

**Design and setting:**

A mixed methods study using online survey data triangulated with qualitative interviews.

**Method:**

Participants were GPs aged <50 years who had left the English Medical Performers List in the last 5 years (2009–2014). A total of 143 early GP leavers participated in an online survey, of which 21 took part in recorded telephone interviews. Survey data were analysed using descriptive statistics, and qualitative data using thematic analysis techniques.

**Results:**

Reasons for leaving were cumulative and multifactorial. Organisational changes to the NHS have led to an increase in administrative tasks and overall workload that is perceived by GP participants to have fundamentally changed the doctor–patient relationship. Lack of time with patients has compromised the ability to practise more patient-centred care, and, with it, GPs’ sense of professional autonomy and values, resulting in diminished job satisfaction. In this context, the additional pressures of increased patient demand and the negative media portrayal left many feeling unsupported and vulnerable to burnout and ill health, and, ultimately, to the decision to leave general practice.

**Conclusion:**

To improve retention of young GPs, the pace of administrative change needs to be minimised and the time spent by GPs on work that is not face-to-face patient care reduced.

## INTRODUCTION

It has been the policy of successive UK governments to address the challenge of the growing healthcare needs of the ageing population by transferring care into the community setting.^1^ In the 10 years prior to 2011, the GP workforce in the UK had an annual average increase of 2.3%.^2^ However, this was only half the rate of other medical specialties.^3^ Patient demand for GP services in England continues to grow, with an estimated 340 million patient consultations per year, an increase of 40 million since 2008.^3^ The UK Department of Health (DH) has set a target that half of all medical graduates entering postgraduate specialty training should go into GP training.^4^ However, despite the longstanding DH policy to increase GP training numbers in England to 3250 per annum, GP recruitment has remained below this target, at around 2700 per annum.^5^ The cost of 5 years’ foundation and GP training programmes is £249 261 per GP.^6^ It is therefore imperative that these highly trained professionals are retained within the UK primary care workforce.

In the 5 years between 2009 and 2014, 45.5% of England’s 12 690 GP leavers were aged <50 years, while 30.6% were aged 50–59 years, and less than one-quarter were aged ≥60 years.^7^ This early loss of GPs is contributing to the GP workforce crisis.^8^ In 2013, Health Education England and NHS England commissioned this mixed methods study to investigate why so many GPs leave the NHS below the age of 50 years.^9^,^10^ This article summarises the main reasons for leaving.

## METHOD

### Study design

A mixed methods study comprising an online survey, triangulated with qualitative interviews, was conducted. To design the survey, the views of 34 GPs were sought. From these, qualitative content analysis was used to identify major categories, which then formed the survey items.^9^

### Survey recruitment

GPs who were aged <50 years and had left the English Medical Performers List between 2009 and 2014 were recruited through articles in *BMA News* as well as direct mailing. Twelve NHS area teams (ATs), between them covering 40% of the population of England, sent invitations to some or all of their early GP leavers. In total, ATs mailed 413 early leavers, and 143 participated in the survey.

### Qualitative interviews recruitment

At the end of the online survey, participants were invited to take part in an interview and 38 survey participants volunteered. Of these, 21 returned signed consent forms. Semi-structured interviews were carried out by telephone, guided by an interview schedule that was developed to complement and extend the survey questions.^10^ Interviews lasting 40–60 minutes were audiorecorded, transcribed verbatim, and all identifying information was removed.

**How this fits in**

Almost half of GP leavers in England are aged <50 years. Key drivers for leaving early relate to changes in the NHS, resulting in loss of professional autonomy, and in overwork, stress, and burnout. General practice in the UK has undergone a series of organisational changes culminating in an increase in day-to-day administrative tasks, which have come to adversely impact the doctor–patient relationship. To improve retention of young GPs in practice, time spent on work that is not face-to-face patient care needs to be minimised.

### Analysis

#### Quantitative

Survey data were analysed using descriptive statistics. Common themes were identified and summarised from the free response survey items using thematic analysis techniques.^11^

#### Qualitative

**Figure 1.**
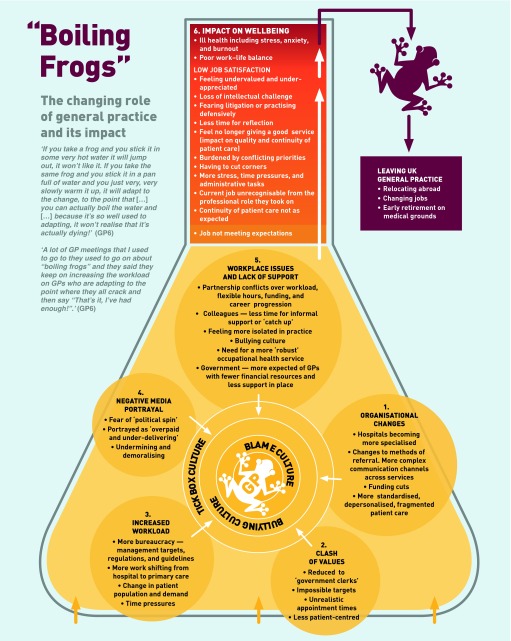
***Boiling frogs: the changing role of general practice and its impact. © Natasha Doran 2016.***

Fieldwork notes contextualised the interview data and detailed summaries of each interview were produced. Thematic analysis was used to generate themes, both within and across the dataset.^11^ The phases of analysis included coding, followed by the identification and clustering of themes and sub-themes, and the production of a descriptive thematic summary. Team members each coded three transcripts, before comparing their analyses for inconsistencies and agreement. Finally the themes and sub-themes were grouped to construct a more interpretative narrative across the dataset and depicted diagrammatically (Figure 1).

## RESULTS

Of the 143 survey responders, 72 (50.3%) were female and 70 (49.0%) male (one unknown). Their median age range was 40–44 years. Forty-six (32.2%) stated that they still work as GPs but outside the EU, seven (4.9%) work as GPs in another part of the EU, and two (1.4%) in another part of the UK (not England). Nineteen (13.3%) do other clinical work, and 22 (15.4%) do non-clinical medical work. Thirteen (9.1%) said that they were full-time parents, and 14 (9.8%) were carers. A further nine (6.3%) had retired voluntarily, and six (4.2%) had retired on medical grounds. The remainder were in non-medical careers.

Of the 21 interviewees, 14 participants were female and seven were male, with an age range of 32–54 years at the time of interview. They had been practising GPs in England for between 2.5 and 20 years; their ages when they left general practice in England ranged from 29–50 years. Interviewees represented a maximum variation sample in terms of age, number of years as practising GPs, and geographical location.

At interview, five participants were working as GPs abroad; five had either retired early on medical grounds or taken jobs that accommodated their illness; eight worked in related fields, such as medical education, the tribunal service, managerial roles, and public health; two decided to focus on raising their families; one worked in a non-medical career.

Although many of the categories in the survey were also identified in the analysis of the qualitative interviews, the inductive and interpretative nature of the qualitative analysis generated a thematic summary that illustrates the complex and overlapping issues causing GPs to leave practice early (Figure 1). The qualitative findings are therefore given primacy here and are supported by relevant statistical evidence from the survey.^9^,^10^

All survey responders indicated that they had left English general practice for multiple reasons:

‘I think it’s so multifactorial, I don’t think there’s any one thing. I think it’s that combination of increased work with decreasing income with high patient expectation with continuous media negativity and no support from the government, just all of those things.’

(GP5)

Although the strength of other social factors such as family issues or attractions to alternative careers were explored during interview and through the online survey as possible determining factors in their decision to leave, participants’ responses were more focused on the impact of their profession on their personal lives, rather than the other way around.

The complex interplay of factors explaining why GPs leave practice early was characterised by the overarching theme ‘The changing role of general practice and its impact’. This is discussed in relation to the sub-themes: organisational changes; clash of values; increased workload; negative media portrayal; workplace issues; and lack of support.

### Organisational changes

Participants described a radically altered working environment caused by an unprecedented increase in organisational changes, many of which were felt to be made without any *‘long-term vision’* (GP19), and for *‘little health gain’* (GP15). Unhappiness with day-to-day life as a GP was indicated by 78.9% of survey responders, in particular, changes to the role of the GP (43.7%):

*‘Cases were getting more complicated, more was being transferred from the responsibility of the hospital to the responsibility of GPs* […]*, I was spending more and more time doing administrative things and less and less time being able to devote my mental attention to the patients in front of me. I just felt more and more stretched.’*

(GP3)

As referral systems became more complex and hospitals more specialised, interviewees experienced a more fragmented and depersonalised healthcare system that was increasingly challenging for them to navigate:

*‘One of the problems with hospital medicine is it’s very fragmented* […]*, so if you sent somebody in with one thing, they have that sorted, but they don’t look at the bigger picture, so they’d come back out and there’d be another thing that was developing so you’d have to refer them to somewhere else, so the fragmented nature of hospital medicine makes general practice quite difficult.’*

(GP4)

### Clash of values

According to participants, continual organisational changes fundamentally altered their professional role to a ‘government clerk’ or a *‘data clerk for public health and for management’* (GP15). The increasing influx of administrative tasks left many feeling professionally compromised as they came to face conflicting priorities in the consulting room. Among survey responders, 55.6% stated that the goalposts were being moved too often and 52.1% disliked the ‘target-driven’ approach to patient care:

*‘You spent more time ticking boxes than you did talking to the patients sometimes* […] *that put more stress on me and I felt it affected my rapport with the patients.’*

(GP2)

For most participants, the introduction of the Quality and Outcomes Framework (QOF) marked a defining point where ‘modern medicine’ became a *‘more target driven culture’* (GP12), or a *‘tick box exercise’* (GP1).

For the majority of participants, attempts to juggle what they saw as *‘impossible targets’* with *‘unrealistic appointment times’* (GP12) detracted from delivering good patient care:

*‘The partner would come in before I started surgery and say, “Oh don’t forget to do all the QOFs* […]*” And that was more important than actually focusing on the patient* […] *With busier and busier surgeries with more and more extras, something has to go and I think what ends up going when you’re under pressure to get all the QOFs and the money in, is the actual patient relationship.’*

(GP11)

### Increased workload

Participants perceived that management targets, regulations, and guidelines impinged on their day-to-day work in general practice, increasing their workload. Fifty per cent of survey responders thought that having too high a non-clinical workload was a factor in their decision to leave practice early, while 83.8% said that aspects relating to pressure of work featured in their decision:

‘The consultation’s length didn’t change, but what you were expected to do in a consultation changed.’

(GP11)

‘I felt I was cutting corners, I felt I wasn’t offering a good service unfortunately.’

(GP6)

The higher administrative workload reduced the time available to spend with their patients, leading to a fundamental change in the doctor–patient relationship:

‘You see it does change the doctor–patient relationship because it changes how you react to people and how you interact with people. I mean it’s obvious stuff, but when you’re really stressed and you’ve still got 15 people to see, you don’t have the time for people, you don’t have the interest.’

(GP11)

The conditions within which doctors were expected to function affected their ability to practise holistic, patient-centred care:

*‘Patients are dissatisfied* […] *because they’re not being given sufficient time to give their history properly and be investigated at the primary care level* […] *there isn’t that reflective quality that allows differential diagnosis, use of time, the use of your personal knowledge of the individual and their social circumstances to be applied.’*

(GP9)

With more work shifting from hospital to primary care, combined with changes in patient population and demand, participants felt increasingly time-stretched. Strategies to cope included staying late at work, taking work home, or changing their appointment times:

*‘I changed my work patterns because I kept getting migraine headaches, because I was getting stressed because of time pressures* […] *I found it very stressful, having patients just waiting, because I was running late on a regular basis.’*

(GP2)

### Negative media portrayal

Factors relating to patients and the media were cited by 63.4% of survey responders. Concerns about media attacks on the medical profession were indicated more frequently (57.0%) than fear of litigation (25.4%) or complaints (17.6%).

Rather than feeling supported in their efforts to meet patient demands, or to cope with the pressures inherent in a high-risk working environment, participants instead felt worn down by negative media representations:

‘I was very conscious of the negative image of general practice in the media and I found it quite stressful.’

(GP3)

For many participants, being portrayed as ‘*overpaid and under delivering*’ was tantamount to ‘*media battering*’:

*‘One of the frustrations is that I think there was definitely a political spin against general practice* […] *It doesn’t help when you’ve had a bad day at work and you come home and watch the 10 o’clock news and you see somebody on the telly saying “Oh these GPs aren’t working very hard and patients can never get appointments”* […] *Just constant criticism in the press about the fact that GPs were getting paid an awful lot of money and they weren’t having to do the out-of-hours and they weren’t working nights and weekends.’*

(GP6)

Not only did participants feel misrepresented by ‘political spin’, but they also felt frustrated that the more positive aspects of their hard work and professionalism went largely unreported:

‘There was never anything positive, never any positive health stories related to the improvement in cardiac mortality, reductions in cancer deaths, earlier diagnosis — any of the positives that we’d achieved were just ignored.’

(GP9)

Being the subject of an ongoing and negative media campaign left many feeling undermined and demoralised:

*‘We were targeted in a completely unsympathetic light* […] *without any recognition of what as a profession we gave to the public really and it did, over time, become very wearing.’*

(GP9)

### Workplace issues and lack of support

Participants described conflicts within their practices over funding, career progression, flexible hours, and workload distribution. These issues within practices were exacerbated by the lack of time for more informal interactions and support among colleagues. Although all participants felt supported during their training and registrar years, once fully qualified they became increasingly isolated in practice:

*‘I did sometimes feel quite isolated at the practice* […] *I think the thing that possibly my training hadn’t prepared me for was sort of feeling like a lone worker in many ways, particularly in comparison to working in a hospital where you were always part of a team.’*

(GP3)

Participants expressed the view that more was being expected of them by government, without the necessary support in place:

*‘I lost my confidence. I lost my faith in the system. I lost my faith in my profession* […] *I think once you’ve lost your confidence, then I don’t think there’s any structure within the profession that helps that come back.’*

(GP4)

Participants described a ‘bullying culture’, which they felt had come to permeate the NHS from the top down:

‘There is a really aggressive, vicious, bullying culture that permeates management in the NHS. That then flows all the way down to whoever your locality middle managers are. It’s a dreadful, awful, bullying culture and to shift from that to a non-overseeing, facilitative, hands-off, trusting culture is ... I don’t know if people are capable of that cultural shift.’

(GP15)

In light of this reported culture of bullying, several interviewees expressed the need for more support, particularly in the form of a more ‘robust’ occupational health service for doctors. Among survey responders, unhappiness with their professional culture was important for 61%, while 44% highlighted the feeling of a loss of autonomy and professional control.

### Impact on job satisfaction and wellbeing

Time pressure and conflicting priorities meant that participants felt that the care they were giving was sub-standard. These pressures, intensified by a perceived *‘blame culture’*, led to disillusionment and a raised anxiety about the risk of making a mistake:

‘I found that I was increasingly anxious about the patients that I was seeing. I think because I was so often quite time-strapped with all the things that I was trying to fit in during the day. But I felt conscious that I was worried that I ran the risk of missing things and that made me really worried and anxious.’

(GP3)

Participants described a series of conditions which they felt contributed to an increasingly pressurised working environment. These included organisational changes resulting in a clash of values and diminishing professional autonomy as health care became more centralised, standardised, and depersonalised; an unprecedented increase in administrative workload; and a lack of support not only from government, but also across services and the wider community due to an ongoing negative media campaign.

This combination of factors led to reduced job satisfaction and ultimately affected wellbeing. In some cases, participants grew to hate their job:

‘I think I got to the point where I hated it and, that’s a really strong word. But I absolutely hated it and I used to wake up on a Friday morning feeling sick at the thought of going in.’

(GP11)

In other cases, it was not so much the job, but ‘*everything around the job*’ that they came to ‘*hate*’, as another participant described:

‘Passionately adoring my work and my patients, I mean, really I can’t tell you how much I miss them. Absolutely loved the actual job, but everything around the job I hated.’

(GP7)

One participant, who had worked in general practice for 18 years and was also an appraiser, described the impact this was having on a number of GPs:

‘There was this kind of malaise growing within the profession that I could see as an appraiser. As GPs got more and more exhausted and burnt out, there was this “I don’t want to know”, there was this disassociation, there was this lack of will to fight to get what patients needed.’

(GP13)

One-third of the survey sample experienced ill health, including stress and anxiety disorder. Burnout was cited by 38.0% of the survey responders, although some participants self-diagnosed the early symptoms of burnout:

‘I don’t think I was medically ill, but I was certainly quite grumpy and I was quite fed up and I just wasn’t enjoying work and I got to the stage when I was driving to work and I used to have this sort of sense of dread the nearer I got to the practice and I thought “Oh no, another day is coming”. I thought this isn’t right, I shouldn’t be feeling like this!’

(GP6)

Others decided to act upon these early-warning signs and leave:

‘Before getting to the point where I really thought I was going to burn out and really hit a very low point mentally and psychologically, I thought actually, I think I recognised those warning signs and I thought it better to go do something different at this point while I still have the wherewithal to go and do it.’

(GP12)

Personal factors were cited by 90.8% of survey responders, in particular, feeling overworked (53.5%), a wish to improve their work–life balance (49.3%), the work being too stressful (43.0%), and lack of enjoyment of the work (41.5%).

Overall, participants felt that their job was not meeting expectations, particularly among GPs who had been in practice for ≥10 years, it was felt that their current job was unrecognisable from the professional role they had initially taken on.

## DISCUSSION

### Summary

Participants had been attracted to GP work in the expectation that it would offer continuity of patient care, professional autonomy, and flexibility in working hours, along with the intellectual challenges inherent in problem solving. However, participants described factors that were both cumulative and multifactorial, leading to their decision to leave practice early in their careers (Figure 1).

The extent and rapidity of organisational changes to the NHS, which had led to an increase in day-to-day administrative tasks and overall workload, were perceived by participants to have fundamentally changed the doctor–patient relationship: the very hallmark of general practice.

Lack of time with patients meant the ability to practise patient-centred continuity of care was perceived to be compromised and, with it, the GPs’ professional autonomy and values, resulting in diminished job satisfaction. Once their job satisfaction had become negatively impacted, the combined pressures of increased patient demand and the negative media portrayal left many feeling unsupported and vulnerable to burnout and ill health, and, ultimately, to the decision to leave general practice.

### Strengths and limitations

GP training, recruitment, and retention in the UK is fast approaching crisis point as more GPs leave the profession at a younger age. This study triangulates interview findings with survey results to provide an in-depth exploration of the reasons why this is happening. Participants were self-selecting and therefore may have had particularly strong views. However, interviewees represented a maximum variation sample in terms of age, number of years as practising GPs, and geographical location.

### Comparison with existing literature

Although current evidence points to an impending crisis in the recruitment and retention of GPs in the UK,^12^–^14^ this is by no means a new phenomenon,^15^–^17^ nor one which is unique to the UK workforce.^18^–^20^ In 2001, a survey carried out by the BMA revealed that one-quarter of GPs wanted to quit,^21^ while a number of surveys, carried out before and since, have continued to monitor GP training, retention, and recruitment, particularly in relation to contractual reforms, job satisfaction, and burnout.^15^,^22^–^26^ Much research has been carried out on factors associated with stress, anxiety, depression, and burnout among doctors in the UK and abroad.^27^–^30^ There has also been a renewed focus in the research literature upon educational initiatives, preventive measures, and therapeutic interventions that could help combat what is perceived to be a growing malaise within the healthcare profession.^31^–^35^

In a recent BMA survey, 80% of 1000 responders rated work pressure as ‘high or very high’, with their main workplace stresses being ‘meeting patients’ demands, lack of time, and excessive bureaucracy’.^36^ In a study looking at motives for early retirement among GPs in the Netherlands, policies related to workload reduction were considered the most useful instruments to control retention and retirement.^37^ The current mixed methods study complements and extends this literature, by showing the cumulative, inter-related, and multifactorial reasons as to why GPs are leaving practice early in their careers.

### Implications for research and practice

The early loss of GPs causes a considerable drain on NHS resources. To improve retention of GPs in practice, NHS leaders need both to minimise the pace of administrative change and to reduce the amount of time spent by GPs on work that is not face-to-face patient care.

For those leaving practice early, exit interviews would help identify specific local as well as more general reasons for their leaving the GP workforce.

Many GPs reported that they had enjoyed direct patient care. Research is needed on how the skills and experience of GPs can most usefully be harnessed, rather than being lost to the NHS.
